# The Relationship between Food Insecurity and Esophageal and Gastric Cancers: A Case-Control Study

**Published:** 2017-06-14

**Authors:** Milad Daneshi-Maskooni, Mahtab Badri-Fariman, Nahal Habibi, Ahmadreza Dorosty-Motlagh, Hashem Yavari, Arvin Kashani, Mostafa Hosseini

**Affiliations:** ^1^ Department of Community Nutrition, School of Nutritional Sciences and Dietetics, Tehran University of Medical Sciences, Tehran, Iran; ^2^ School of Agriculture, Food & Wine, University of Adelaide, Adelaide, South Australia, Australia; ^3^ Department of Clinical Nutrition, School of Nutritional Sciences and Dietetics, Tehran University of Medical Sciences, Tehran, Iran; ^4^ Department of Epidemiology and Biostatistics, School of Public Health and Institute of Public Health Research, Tehran University of Medical Sciences, Tehran,

**Keywords:** Food insecurity, Esophagus, Stomach, Cancer, Iran

## Abstract

**Background:** Food insecurity is defined as the limited or uncertain availability of enough food for permanent active and healthy life. Upper gastrointestinal (GI) cancers (esophagus and stomach) are one of five most common cancers in Iran. This study aimed to determine the association of food insecurity and upper GI cancers in newly diagnosed patients.

**Study Design:** Case-control

**study Methods:** Overall, 120 patients with upper GI cancers as cases and 120 patients with orthopedic, earnose-throat (ENT), and neurologic diseases as controls were recruited from Imam Khomeini Hospital, Tehran, Iran in 2013. The patients were newly diagnosed using endoscopy or imaging or biopsy methods. They were individually matched for age, sex, and residential area. The general and United States Department of Agriculture (USDA) household food security questionnaires were completed. The univariate and multivariate conditional logistic regression tests were applied using the Stata 11SE statistical software.

**Results:** The food insecurity prevalence was 69.2% and 43.3% in cases and controls, respectively. Food insecurity, low economic level and family history of cancer were significantly associated with cancer (*P*<0.05).

**Conclusions:** Food insecurity was one of the important risk factors for upper GI cancers that health care providers should consider it.

## Introduction

The concept of food security was attended in the early years of 1970s over the food crisis and several‏‏ ‏studies‏‏ ‏evaluated its‏‏ ‏various perspectives^1^. According to the 1996 World Food Summit (WFS), food security exists when all ‎people, at all times, have physical and financial access to adequate, safe and nutritious food to ‎achieve their dietary essentials and food inclinations for a dynamic and healthy life^2^. Food secure peoples do not live in hunger or dread of starvation^3^. Then again, food insecurity as indicated by the United States Department of Agriculture (USDA) is a circumstance of constrained or unverifiable availability of ‎nutritionally satisfactory and safe foods or restricted or indeterminate ability to obtain acceptable foods in ‎socially worthy ways^4^. Therefore, four columns of food security are availability, access, utilization, and stability^3^.

Food‏ insecurity with‏ inadequate intakes‏ of essential nutrients may prompt mental and behavioral‏ dysfunction‏ in children and‏ adults and reduce resistance to diseases. Inability to‏ purchase‏ nutritious and adequate food and its mental and emotional stresses affect human health detrimentally, and even result in aggravation of other underlying diseases caused by other risk factors. Food insecurity is indirectly in relationship with poor health status, certain chronic diseases, adulthood obesity, hypertension, abnormal blood glucose levels, dyslipidemia, increasing levels of C‐reactive protein, depression, decreasing ability in daily activities, heart failures, gum disease, asthma and cognitive decay^5^. The food insecurity and poor health status can prompt each other. Continuous and periodic food deficiencies may be the more effectively trigger health challenges that can augment, in ‎turn, food insecurity^6^.

Increasing food insecurity over the past decade, particularly in developing countries was because of economic levels decline that raise fuel and food costs^7^. The food insecurity prevalence was 14.7% in America, 10% in Canada, 50% in Malaysia, 11% in Indonesia, 35% in the Philippines, 70% in Bolivia, 73% in Burkina Faso, 5.7% in Finland, 43% in Pakistan, and nearly 50% in Iran^5^.

Cancer as a debilitating disease shortens the lifetime and contributes to various individual and social harms‏. The importance of cancer as a main cause of death is increasing. Cancer was ranked as the second cause of mortality after cardiovascular diseases in the world^8^. The upper gastrointestinal (GI) tract (esophagus and stomach) cancers were one of the five most common cancers in Iran^9^.

Attention to the effects of food insecurity on health and social and psychological consequences, evaluation of its perspectives is essential^10^. In a study among cancer patients, the food insecurity prevalence was significantly higher than general peoples^11^. The relationship between food insecurity and some diseases, including obesity/overweight, diabetes, depression, and other mental and physical illnesses has been demonstrated^5^.

Considering the increasing food insecurity and upper GI tract cancers in Iranian population, possibility of relationship, and no previous studies, we determined the association of food insecurity and upper GI tract cancers in newly diagnosed patients.

## Methods

### Study Participants

This case-control study was conducted at Imam Khomeini Hospital‏ of Tehran, Iran, from Jan-Dec 2013. The case group included 120‏ newly diagnosed patients with‏ upper GI tract cancers admitted in the surgical ward of Central Institute of Cancer. The control group was 120 non-cancerous patients at one of the Orthopedics, Ear-Nose-Throat (ENT) and Neurology Departments. The age, sex and residential status (Tehran, around of Tehran, and other cities) were matched between two groups.

### Ethics statement

Ethical clearance as a master’s thesis was obtained from the Ethics Committee of Tehran University of Medical Sciences (RegNo.139). Before the data collection, a written informed consent form was taken from the patients.

### Data Collection

All of the participants completed the general and USDA household food security status questionnaires by interviewing. The Persian version of USDA household food security questionnaire and the general questionnaire were used already^12, 13^.

The value of Cronbach's alpha for USDA household food security survey model (HFSSM) was 0.80 in US^4, 14^ and 0.72 in Iran^12^. Furthermore, the adapted model of HFSSM in Isfahan survey gave internally valid household-level measures of food insecurity among adults and children. The scattering of items over a notable range of severity alongside passable item-fit statistics showed high average item difference and good fit to the Rasch measurement model. The other studies in several countries including Bolivia, Brazil, Colombia, Mexico City, Trinidad and Tobago, Ecuador, and Canada have been conducted to validate the HFSSM. Their results were similar in confirming the proportionality of the HFSSM to measure the severity of household food insecurity using different internal validity tests and methods^14^.

The questions of general questionnaire included age, sex, residential area, economic ‎levels, ethnicity, education levels, health insurance, history of ‎GI ulcer, family ‎history of cancer, body mass index (BMI), family size, number of ‎children, marital ‎status, history of smoking at ‎‎6 months‎‏ consecutively‎‏, and history of ‎specific ‎diseases (including diabetes, heart disorders, hypertension).

The inclusion criteria were patients with upper GI cancers (stomach, esophagus, or both), diagnosis duration less than 6 months, and age more than 20 yr. The exclusion criteria were disability to answer, cooperate or remember (Alzheimer, Amnesia, etc.) and psychotic diseases.

The methods of diagnosing upper GI cancers were endoscopy, imaging, or biopsy/cytology depending on the doctor opinion.

Before beginning, a pilot study was conducted among 19 newly diagnosed cancer patients to clarify the research perspectives.

The economic status was determined based on nine items: car ownership, house ownership, washing machine, LCD TV, dishwasher, side-by-side refrigerator, handwoven carpets, computers/laptops, and microwave oven. The three economic levels were low/poor (< 3 items), moderate (4-6 items) and good/wealthy (>7 items)^13^.

According to the 18-item USDA food security status questionnaire, food security is classified into four groups: food secure, food insecure without hunger, food insecure with moderate hunger, and food insecure with severe hunger. In this study, participants were divided into two food secure and food insecure groups. The scoring range of food security was 0 - 18 according to the numbers of positive responses (food secure: 0-2, and food insecure: 3 - 18)^4^.

Weight and height were obtained from medical records and BMI was calculated (BMI <18.5 kg/m^2^, 18.5-24.99 kg/m^2^, 25.0-29.99 kg/m^2^, and ≥30 kg/m^2^ as underweight, normal, overweight, and obese, respectively).

Data were analyzed using the Stata11SE statistical software. The conditional logistic regression models were used to identify the risk factors of upper GI cancers. The individually matched factors were age, sex, and residential area. A *P*-value <0.05 was considered significant statistically. The significant factors in univariate model were entered to multiple conditional logistic regression to identify probable risk factors for upper GI cancers.

## Results

Participants were totally 240 patients (144 men, 96 women) aged 71.1 ± 32.6 yr. Patients with gastric, esophageal or both cancers were 79 (55 men and 24 women), 30 (10 men and 20 women) and 11 (7 men and 4 women) and patients with neurological, orthopedic and ENT diseases were 33 (13 men and 20 women), 51 (35 men and 16 women) and 36 (24 men and 12 women), respectively.

The residual areas of 27.5%, 11.7%, and 60.8% of any groups were the center of Tehran, the around of Tehran, and the other cities, respectively.

The mean ± standard deviation (SD) of age, weight, and height in cases and controls were 60.23±11.48 yr and 60.42±11.99 yr, 59.71±12.53 kg and 67.67±9.72 kg, and 163.19 ±9.64 cm and 166.96 ± 8.18 cm, respectively.

The food insecurity prevalence was 69.2% in cases and 43.3% in controls. The mean ±SD of food security score in cases and controls were 3.297±2.437 and 1.848±2.081, respectively.

According to the univariate conditional logistic regression model, the food security, economic status, education status, ethnicity, health insurance status, GI ulcer history (gastric or duodenal), family history of cancer, body mass index and family size differed significantly between two groups. The majority of cancerous patients were in food insecurity, lower economic levels, non-Fars ethnicity, illiterateness, family size≥3 peoples, GI ulcer history or family history of cancer, and health insurance coverage status. The other factors, including marital status, smoking history, and history of specific diseases did not have significant differences between the two groups (Table 1).

**Table 1:** Comparing various factors between patients with upper GI cancers and non-cancerous controls using conditional logistic regression (individually matched for sex, age, and residential area)

**Factors**	**Cases**		**Controls**		**Percent**	**OR (95% CI)**
	**Number**	**Percent**	**Number**	***P*** ** value**		
Food security status						
Secure	37	30.8	68	56.7	1.00	
Insecure	83	69.2	52	43.3	2.93 (1.72, 4.98)	0.001
Economic level						
Medium/High	24	20.0	41	34.2	1.00	
Low	96	80.0	79	65.8	2.07 (1.15, 3.72)	0.014
Ethnicity						
Persian	22	18.3	68	56.7	1.00	
Non-Persian	98	81.7	52	43.3	5.82 (3.24, 10.47)	0.001
Level of education						
Literate	59	49.2	77	64.2	1.00	
Illiterate	61	50.8	43	35.8	1.85 (1.10, 3.10))	0.020
Having health insurance						
No	6	5.0	20	16.7	1.00	
Yes	114	95.0	100	83.3	3.8 (1.46, 9.83)	0.006
History of gastric ulcer						
No	100	83.3	111	92.5	1.00	
Yes	20	16.7	9	7.5	2.46 (1.07, 5.66)	0.033
Family history of cancer						
No	76	63.3	96	80.0	1.00	
Yes	44	36.7	24	20.0	2.31 (1.29, 4.14)	0.005
Body mass index (kg/m^2^)						
Normal weight (18.5-25.0)	61	50.8	74	61.7	1.00	
Under weight (<18.5)	23	19.2	2	1.6	13.72 (3.11, 60.52)	0.002
Overweight/obesity (>25.0)	36	30.0	44	36.7	0.94 (0.54, 1.65)	
Family size						
1-2 persons	31	25.8	56	46.7	1.00	
≥3 persons	89	74.2	64	53.3	2.51 (1.45, 4.32)	0.001
Marital status						
Married	110	91.7	107	89.2	1.00	
Single/widowed/divorced	10	8.3	13	10.8	0.74 (0.31, 1.77)	0.512
History of smoking for ≥6 months						
No	85	70.8	94	78.3	1.00	
Yes	35	29.2	26	21.7	1.48 (0.82, 2.67)	0.183
History of specific diseases						
No	78	65.0	70	58.3	1.00	
Yes	42	35.0	50	41.7	0.75 (0.44, 1.27)	0.289

Ultimately, risk factors of upper GI cancers were food insecurity, family history of cancer, and lower economic levels (Table 2).

**Table 2:** Risk factors for upper GI cancers using multiple conditional logistic regression (individually matched for sex, age, and residential area)

**Factors**	**Cases**		**Controls**		**Percent**	OR* (95% CI)
	**Number**	**Percent**	**Number**	***P*** ** value**		
Food security status						
Secure	37	30.8	68	56.7	1.00	
Insecure	83	69.2	52	43.3	2.57 (1.41, 4.66)	0.002
Economic level						
Medium/high	24	20.0	41	34.2	1.00	
Low	96	80.0	79	65.8	2.42 (1.23, 4.76)	0.010
Family history of cancer						
No	76	63.3	96	80.0	1.00	
Yes	44	36.7	24	20.0	1.98 (1.03, 3.80)	0.040

^*^Adjusted for economic ‎levels, ethnicity, education levels‎, health ‎insurance, history of GI ‎ulcer, family history of ‎cancer, BMI, family size

## Discussion

69.2% of the patients with upper GI cancers and 43.3% of the controls had food insecurity. The food insecurity, family history of cancer, and lower economic level were significant risk factors for upper GI cancers. In only a cross-sectional study among 115 cancerous patients (various types of cancer) in Kentucky, with and without hunger food insecurity prevalence were 9.6% and 7.8%, respectively. These rates were more than in general peoples of Kentucky^11^.

The current study was the first on the food security status of cancer patients in Iran. The average food insecurity prevalence in the past studies on general population in Iran was about 45%^13^. Moreover, the food insecurity prevalence in the United States between 1995 and 2012 varied from 10.1% to 14.7%^5^. The food insecurity prevalence in other studies on general peoples using USDA household food security questionnaire was reported less than this study^3, 5, 15^. These differences may be the result of the different methods and participants.

Food insecurity, family history of cancer and lower economic level were the most relevant risk factors for upper GI tract cancers. The odds ratio of cancer in peoples with food insecurity, low economic level, and family history of cancer were 2.57, 2.42, and nearly 2 times, respectively.

The grades of food insecurity are concern about adequate food and reducing the size and number of meals, decreasing the size and number of meals, weight losing, feeling hunger, and reducing the size or number of the children’s meals. Recently, the paradox of food insecurity and health conditions has been concentrated in literatures. The food insecure populations often have the limited available budget, leads to purchase and consume cheaper and nutritionally insufficient foods (e.g., high dense calorie and fat, lower fruits and vegetables, lower quality, lower micronutrients) that might contribute to obesity, and an increased susceptibility to chronic illnesses including cancer, type 2 diabetes, depression and other medical condition such as acne, dyspepsia and so on^5^. Insecure food insecurity creates anxiety and worry, because of the social and biological importance of food that increases the probability of growing usual mental disorders. Besides, anxiety and worry can provoke encountering food insecurity and using nutritionally inadequate and high-calorie foods (i.e., comfort meals)^5, 16-18^. Therefore, food insecurity may increase risk of nutrient deficiency and consequently the risk of cancer.

The risk of adenocarcinoma and esophageal squamous cell carcinoma increases with low socioeconomic level according to previous studies in Iran and other countries^19-21^. In coastal areas of Iran, the incidence of esophageal and gastric cancer was lower in high-income peoples^22^. Furthermore, food insecurity was associated with the lower economic levels^3, 15^. The income level is an important factor to access to adequate food in the society and peoples with good economic status have more choices of food^23^. Recently, Iranian politicians and policymakers have focused specially on resistance economy to improve the economic status^24^.

Family history of cancer was a risk factor for gastric cancer and larger masses^25^. In addition, family history of digestive tract cancer was significantly associated with the higher risk of gastric cancer (cardia and non-cardia), but did not increase the risk of esophageal cancer. Family history of breast cancer was considerably associated with the risk of both esophageal and gastric cardia cancers^26^. The family history of the mouth, oral cavity, pharynx and stomach cancers increases risk of esophageal cancer significantly^27^. In Iran, family history of esophageal cancer in the first-degree relatives was associated with the higher risk of esophageal cancer^28^. In contrast, neither the family history of esophageal cancer, nor the family history of stomach or other digestive tract cancers in the first-degree relatives did increase risk of the esophagus carcinoma. However, the risk of stomach cardia adenocarcinoma is moderately increased with a family history of gastric cancer in the first-degree relatives^29^. This inconsistency may be, because of the differences between populations and some associated conditions.

The other study variables including ethnicity, education levels, health ‎insurance, history of GI ‎ulcer, BMI, family size, marital status, history of ‎smoking, and history of ‎specific ‎diseases were not significantly associated with upper GI cancers.

Unlike this study, the BMI and risk of upper GI cancers were inversely related in several previous literatures^30, 31^. The data on weight and BMI of patients before cancer incidence were not accessible.

The demographics of upper GI cancers have been altering drastically on the recent decades. Its occurrence differs broadly based on socioeconomic status, BMI, and history of GI ‎ulcer, smoking, and underlying diseases. Comprehending the etiology and epidemiology of upper GI cancers will prompt interventions for averting and screening in high-risk populaces^32^.

Some risk factors of upper GI cancers including *Helicobacter pylori* infection, dietary pattern, being overweight or obese before of cancer incidence, stomach lymphoma, gastroesophageal reflux disease, Barrett’s esophagus, achalasia, tylosis, Plummer-Vinson syndrome, workplace exposures, injury to the esophagus, and few viral infections, could not be verified in this study.

The selective revealing or suppression of information by subjects may be as a potential reporting bias, that the statistical methods addressed it partly.

The strengths of the study are case-control design, the nobility, obtaining an informed consent form (in Persian) from all the participants, selecting the newly diagnosed cancer patients, individually matching for age, sex, and residential area, conducting a pilot study before the beginning, and no bar to receiving the other health care services of the center.

The limitations of the study are self-reporting, limited types of upper GI cancers, slow patient recruitment because of the eligibility criteria, a single center, and lack of cooperation of some patients in the end, which lead to patients be replaced.

The implications of the work for practice and research are the selecting controls, the difficulty of obtaining reliable information about an individual’s exposure status over time, the difficulty of identifying the newly diagnosed cancer patients, poor mental condition and impatience of cancer patients, and expecting of some patients for financial supports.

## Conclusions

The food insecurity prevalence in patients with upper GI cancers was high. The food insecurity, poor economic status, and family history of cancer may be some risk factors for upper GI cancers. The noticing to comprehending of food insecurity effects on health reduces the burden of it. Even so, other studies are still needed to find association of food insecurity with other cancers or health problems. It is necessary to assess the food insecurity prevalence in different provinces of Iran and investigate diseases associated to it in future studies to depict an obvious illustration of food insecurity.

## Ethical Considerations

Ethical issues (Including plagiarism, informed consent, misconduct, data fabrication and/or falsification, double publication and/or submission, redundancy, etc.) have been completely observed by the authors.

## Acknowledgment

Tehran University of Medical Sciences (RegNo.139) supported this master’s thesis. Cooperation of participants and Cancer Research Center is acknowledged.

## Conflict of interest statement

The authors declare that there is no conflict of interest.

## Funding

None.

## Highlights

- The food insecurity prevalence is notably higher in patients with upper GI cancers.
- The proof of upper GI cancers increases with lower food security and economic levels.
- The family history of cancer increases upper GI cancers.
